# Paroxysmal pain as the only presentation of focal epilepsy

**DOI:** 10.1002/ccr3.3143

**Published:** 2020-07-20

**Authors:** Daniela Garcez, Joana Marques, Mariana Fernandes, John Peter Foreid

**Affiliations:** ^1^ Department of Neurology Instituto Português de Oncologia Francisco Gentil de Lisboa Lisboa Portugal; ^2^ Neurophysiology Laboratory Instituto Português de Oncologia Francisco Gentil de Lisboa Lisboa Portugal

**Keywords:** glioblastoma, ictal pain, insula, painful seizure, parietal operculum

## Abstract

Painful seizures can be the only feature of focal epilepsy. Its localization on a limb favors the epileptic etiology, while abdominal or cephalic paroxysmal pain is not so specific. Painful somatosensory seizures arise from operculo‐insular cortex.

## Graphical abstract

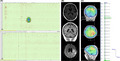

## 1 BACKGROUND

A 55‐year‐old man with left temporo‐insulo‐parietal glioblastoma was admitted because of abrupt excruciating pain in the right arm and ipsilateral face. EEG showed a left central frontal rhythmic activity, and complaints were controlled with antiepileptic drugs. Paroxysmal pain is an unusual manifestation of focal seizures and can be the only symptom.

Ictal pain is a rare manifestation of focal seizures, with a frequency ranging from 0.2% to 2.8% in patients with epilepsy.^1^ Classically, it is categorized according to its localization as cephalic, abdominal (visceral or pneumogastric), or unilateral somatosensory pain.^2^ The last is usually striking and can be described as a burning, stabbing, prickling, throbbing, or tearing sensation. In most cases, they are accompanied by other motor, sensory, or behavioral features, which can denote their ictal origin.^3^ When it occurs in isolation, seizures are often misdiagnosed and patients can go through unnecessary diagnostic procedures and inadequate treatment. While most patients who present with paroxysmal headache or abdominal pain do not have an epileptic cause in their origin, the localization of paroxysmal pain on a limb is more suggestive of an underlying epileptic cause.^1^

## 2 CASE PRESENTATION

**FIGURE 1:**
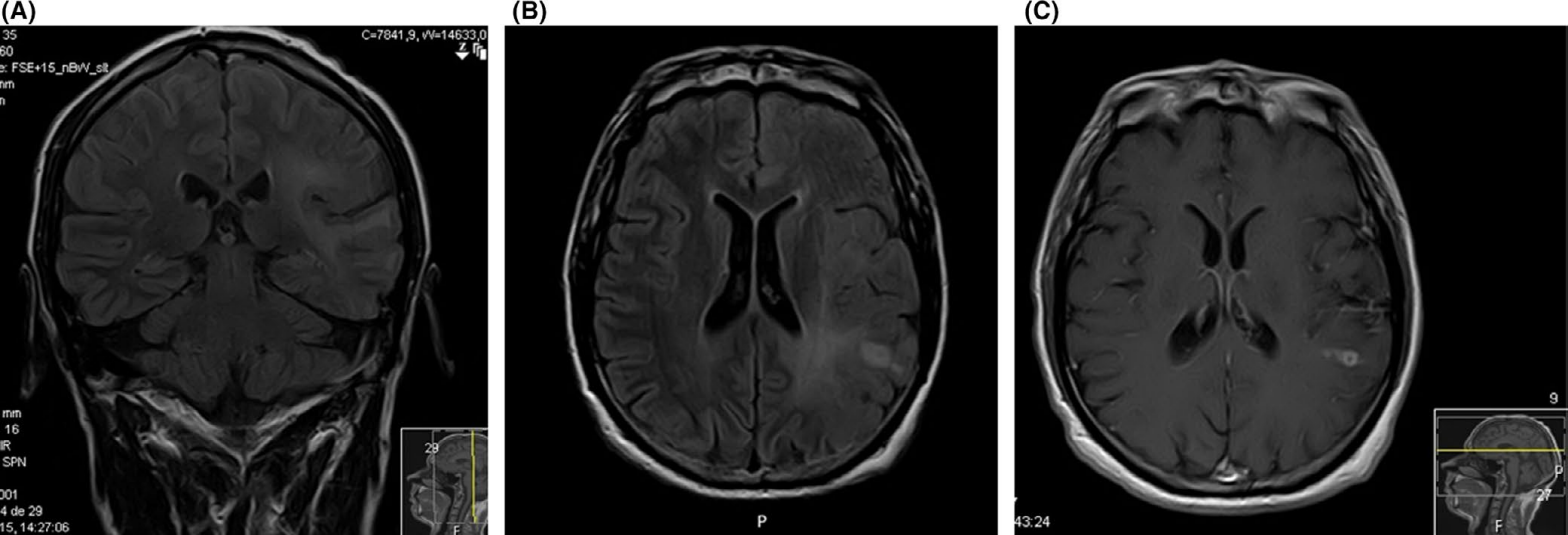
(A) Coronal flair, (B) axial flair, and (C) axial T1 + gad brain MRI sequences showing a left temporo‐insulo‐parietal glioblastoma

**FIGURE 2:**
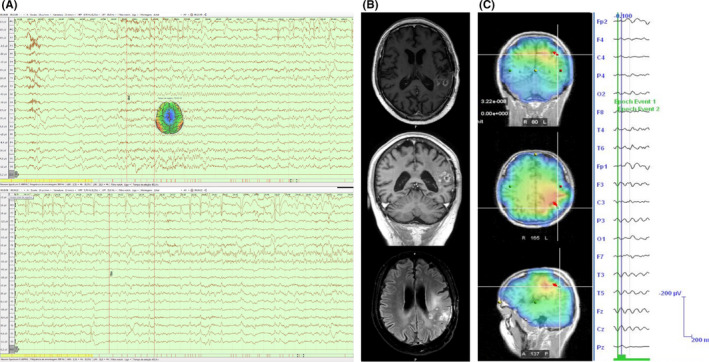
(A) EEG of painful seizure, which starts with diffuse attenuation of electrogenesis, followed by rhythmic theta activity (left fronto‐centro‐parietal and midline) with progressive slowing until the end of the painful episode; (B) Brain MRIs (T1 + gad and flair sequences) showing local tumor recurrence; (C) sLoreta (fast Fourier transform approximation) for the first seconds of the rhythmic theta activity. Inverse solution with a left parietal localization. ASA 4.7 software with standard MRI and electrode digitalization for the 10/20 system

A 55‐year‐old man was diagnosed with a left temporo‐insulo‐parietal left tumor after developing focal cognitive seizures with expressive aphasia or complex visual hallucinations (Figure 1). He underwent partial tumor resection resulting in persistent conduction aphasia, characterized by a slight difficulty repeating and naming everyday objects, with literal paraphasic errors. The histopathology diagnosis was compatible with a glioblastoma. Due to increase in seizure frequency, characterized by aphasic exacerbation afterward that included inability to understand language, he was medicated with successive and incremental doses of levetiracetam and sodium valproate until complete response with 3000 mg/day and 2000 mg/day, respectively. He started treatment with chemotherapy and radiotherapy according to STUPP protocol (temozolomide concomitant with radiotherapy and subsequent six cycles of adjuvant temozolomide), with an initial good radiological response. While undergoing his first adjuvant chemotherapy cycle, he was admitted in the emergency room, desperately screaming because of acute and excruciating pain on the right arm and ipsilateral face, described as shock‐like and stabbing. Each episode lasted a few seconds but they had been repeating in cluster for over an hour. General blood analyses were unremarkable; the electroencephalography showed a left central frontal rhythmic activity during the pain episodes (Figure 2). The add‐on of intravenous lacosamide (bolus of 200 mg) resolved the painful complaint in a few minutes, and he continued subsequent treatment with 200 mg of lacosamide every 12 hours. The brain MRI performed a few days later showed local tumor recurrence (Figure 2). After being discharged, he continued treatment regimen with lacosamide (400 mg/day), levetiracetam (3000 mg/day), and sodium valproate (2000 mg/day), along with oral temozolomide and dexamethasone. The follow‐up brain MRIs showed a very mild tumor growing, concurrent with the development of the right hemiparesis with brachial predominance. Although he remained seizure‐free, we had to reduce sodium valproate (1200 mg/day), because of the hepatic and hematologic toxicity induced by chemotherapy. Unfortunately, the patient died, 7 months after hospital discharge, because of infectious complications.

## 3 DISCUSSION

This case refers to a man with a temporo‐insulo‐parietal glioblastoma, partially removed, undergoing active oncological treatment and with controlled symptomatic focal epilepsy who presented to the hospital with sudden excruciating somatosensory pain. The pain was restricted to the right arm and face, which locates the site of the neurological dysfunction to the left cortical hemisphere. A clue to identifying its nature as epileptic may be its relatively brief and abrupt stereotypical paroxysmal occurrence. Although ictal pain is often associated with other seizure symptoms, it can be the only manifestation of focal epilepsy. Among patients with ictal pain, those who report it to their limbs are more likely to have an epileptic cause.^1^ The presence of positive symptoms points to an irritative rather than destructive disturbance but in this context the most likely cause would be a tumor relapse. There is an inverse relationship between seizure prevalence and tumor growth rate,^4^ and actually, in our case, although we were dealing with a highly aggressive tumor, the follow‐up brain MRIs did not show a fast‐growing rate, which could allow enough time for the tumor cells to re‐organize, vascularize, and develop mechanisms necessary for epileptogenesis.^4^ Under some pathophysiological conditions, operculo‐insular lesions may produce location‐specific painful epileptic seizures. Functional imaging studies have identified several cortical areas activated by painful stimuli, referred as “pain matrix,” including, among others, the primary somatosensory area, the supplementary motor area, the insula, and the anterior frontal or the posterior parietal cortices.^5^ Some cases of ictal pain with foci originating in the frontal, parietal, and temporal regions have been described with surface EEG.^4^, ^6^ However, data from functional cortical mapping by using direct cortical stimulation suggest that the origin of painful somatosensory seizures arises solely from the medial part of parietal operculum or the posterior and upper part of the insular cortex.^7^, ^8^

Taking into account the cortical mapping studies and the localization of our patient's glioblastoma, we suggest that the painful seizure has a left operculo‐insular origin and that the rhythmic activity captured on the surface EEG represents propagation to the primary parietal cortex.

The optimal antiepileptic therapy in patients with brain tumors and epilepsy remains to be defined. We decided to add lacosamide because it has a favorable pharmacokinetic profile, including a lack of induction or inhibition of hepatic enzymes, low protein binding, and low potential for drug interactions. Such characteristics make lacosamide an interesting therapeutic option for patients who are undergoing antineoplastic treatments.

Paroxysmal contralateral pain can be the only manifestation of epileptic seizures with operculo‐insular cortex involvement. This knowledge is useful to avoid misdiagnosis and to prompt appropriate management with antiepileptic drugs.

## CONFLICT OF INTEREST

None declared.

## AUTHOR CONTRIBUTION

DG: wrote the manuscript. JM and MF: evaluated the patient and revised the manuscript. and JF: carried out the EEG assessment. All authors approved the final version to be published.
